# Acute pharmacogenetic dystonic reactions in a family with the *CYP2D6* *41 allele: a case report

**DOI:** 10.1186/s13256-021-03022-x

**Published:** 2021-08-19

**Authors:** Darice Y. Wong, Brent L. Fogel

**Affiliations:** 1 Department of Neurology, David Geffen School of Medicine , University of California Los Angeles, 695 Charles E. Young Drive South, Gonda Room 6554A, Los Angeles, CA 90095 USA; 2 Clinical Neurogenomics Research Center, David Geffen School of Medicine, University of California Los Angeles, Los Angeles, CA USA; 3 Department of Human Genetics, David Geffen School of Medicine, University of California Los Angeles, Los Angeles, CA USA

**Keywords:** CYP2D6, Pharmacogenetic, Ondansetron, Metoclopramide, Prochlorperazine, Dystonia, Neurogenetics

## Abstract

**Background:**

Dystonia is a known neurological complication of certain medications; however, the mechanism behind such effects is often undetermined. Similarly, the clinical pharmacogenomic effects associated with various alleles of the cytochrome P450 family of proteins, and their role in acute dystonic reactions, are also presently unknown.

**Case presentation:**

We describe a woman presenting with acute dystonic reactions to ondansetron, prochlorperazine, and metoclopramide followed by persistent focal dystonia. A similar family history was reported in her siblings and her father to prochlorperazine, drugs all metabolized by the cytochrome P450 2D6 (*CYP2D6*) enzyme. Pharmacogenomic testing indicated the patient was heterozygous for the intermediate metabolizer *41 allele (*CYP2D6* 2988G>A, NM_000106.6:c.985+39G>A, rs28371725). Her father was homozygous for this *CYP2D6* *41 allele, and consequently, her siblings were obligate carriers.

**Conclusions:**

The metabolism of ondansetron, metoclopramide, or prochlorperazine in patients with the *41 *CYP2D6* allele has not been studied. In this family, clinical evidence implicates the *41 *CYP2D6* allele as causing extrapyramidal adverse pharmacologic reactions. Patients with a family history of medication-induced dystonia involving these medications should be considered for pharmacogenomic testing, and patients carrying the *41 *CYP2D6* allele should consider reduction or avoidance of CYP2D6-mediated medications to minimize the potential risk of adverse extrapyramidal effects.

## Background

Acute dystonia, a disorder of abnormal muscle tone leading to spasms and/or abnormal posture, can occur after exposure to certain medications, typically dopamine receptor antagonists [1]. Symptoms can appear following the initial dose, can be focal, segmental, or generalized, and often involve the ocular muscles, face, jaw, neck, tongue, and trunk [1]. In severe cases, this can be life threatening. The mechanism behind these effects is not fully understood but, in some cases, may be precipitated by impairments in drug metabolism, such as by the cytochrome P450 family of proteins. However, the full clinical spectrum of pharmacogenomic effects associated with various alleles of the cytochrome P450 proteins, and their contribution to complications such as dystonia, is also presently unknown.

## Case presentation

We describe a 39-year-old white non-Hispanic woman of Ashkenazi descent who presented with focal dystonia characterized by bilateral blepharoclonus on eyelid closure, sequelae from a prior right-sided Bell’s palsy, and mildly elevated tone in both arms. She had suffered an acute dystonic reaction immediately following intravenous administration of ondansetron in her early twenties, including retrocollis and oculogyric crisis, as well as a later episode of acute akathisia in response to prochlorperazine, and, most recently, akathisia occurring within 1 hour of 10 mg of metoclopramide being given orally, followed by persistence of the current symptoms. There is a family history of similar reactions on the paternal side. Her father had severe akathisia in response to prochlorperazine, as did her brother. Her sister had also experienced akathisia after an epidural during pregnancy.

This history and presentation were concerning for a pharmacogenomic effect, supported by the observation that these drugs are all metabolized by cytochrome P450 2D6 (*CYP2D6*), expressed in the liver and central nervous system. This enzyme is known to interact with a large number of clinical drugs and has a known relationship with extrapyramidal syndromes related to antipsychotics [2].

**Fig. 1:**
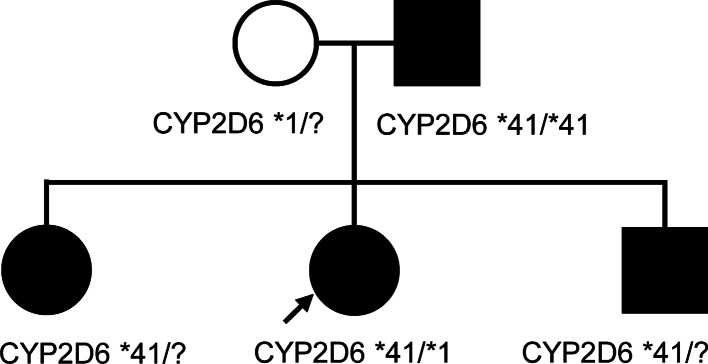
Pedigree. Proband (arrow) and patient genotypes are indicated. ? = not determined, listed genotype inferred from known familial relationships

Pharmacogenomic testing was performed for *CYP2D6* and indicated the patient was heterozygous for the *41 allele (*CYP2D6* 2988G>A, NM_000106.6:c.985+39G>A, rs28371725), an intronic polymorphism associated with aberrant splicing of *CYP2D6* leading to the skipping of exon 6 with a corresponding reduction in activity [3]. This variant is responsible for the intermediate metabolizer phenotype in the majority of people of European descent [4]. Testing of other family members indicated her father was homozygous for the *CYP2D6* *41 allele in the gene, and consequently, her sister and brother were obligate carriers (Fig. 1).

## Conclusions

There is limited information on the relationship between the *41 allele and dystonia. In one study of the drug risperidone, intermediate metabolizers with the *41 allele showed increased levels of the drug and its metabolites (nearly twofold versus poor, or *CYP2D6* loss-of-function metabolizers, which show threefold) with an increased risk of developing dystonia and parkinsonism adverse effects [5]. There are presently no studies assessing the *41 intermediate metabolizer allele in patients taking ondansetron, metoclopramide, or prochlorperazine; however, patients homozygous for inactive loss-of-function alleles (*4 or *5) in *CYP2D6* have been reported with acute dystonic reactions to metoclopramide [6, [7]. In this family, the available clinical evidence points to the *41 allele being the cause of their extrapyramidal adverse pharmacologic reactions despite showing reduced activity rather than being loss-of-function. Future studies should address correlations between genotype, symptom onset, and/or severity with dosage. In this family, we cannot exclude additional genetic or environmental modifiers affecting the overall metabolism of these medications. For example, it has been proposed that elevated estrogen levels during pregnancy can exacerbate dystonic reactions to metoclopramide in poor *CYP2D6* metabolizers [7], which may have contributed to the dystonic reaction seen in our patient’s sister.

We recommend that patients with a family history of medication-induced dystonia involving ondansetron, metoclopramide, or prochlorperazine be considered for pharmacogenomic testing, and that patients carrying the *41 *CYP2D6* allele should consider reduction or avoidance of CYP2D6-mediated medications to minimize the potential risk of adverse extrapyramidal effects.

## Data Availability

Data generated during the current study are not publicly available due to patient privacy but are available from the corresponding author on reasonable request.
